# Effect of acute postsurgical pain trajectories on 30-day and 1-year pain

**DOI:** 10.1371/journal.pone.0269455

**Published:** 2022-06-10

**Authors:** Saria S. Awadalla, Victoria Winslow, Michael S. Avidan, Simon Haroutounian, Thomas G. Kannampallil

**Affiliations:** 1 Division of Epidemiology & Biostatistics, School of Public Health, University of Illinois at Chicago, Chicago, Illinois, United States of America; 2 Department of Anesthesiology, Washington University School of Medicine, Saint Louis, Missouri, United States of America; 3 Institute for Informatics, Washington University School of Medicine, Saint Louis, Missouri, United States of America; Henry Ford Hospital Systems & Outcomes Research Consortium, Cleveland Clinic, UNITED STATES

## Abstract

Untreated pain after surgery leads to poor patient satisfaction, longer hospital length of stay, lower health-related quality of life, and non-compliance with rehabilitation regimens. The aim of this study is to characterize the structure of acute pain trajectories during the postsurgical hospitalization period and quantify their association with pain at 30-days and 1-year after surgery. This cohort study included 2106 adult (≥18 years) surgical patients who consented to participate in the SATISFY-SOS registry (February 1, 2015 to September 30, 2017). Patients were excluded if they did not undergo invasive surgeries, were classified as outpatients, failed to complete follow up assessments at 30-days and 1-year following surgery, had greater than 4-days of inpatient stay, and/or recorded fewer than four pain scores during their acute hospitalization period. The primary exposure was the acute postsurgical pain trajectories identified by a machine learning-based latent class approach using patient-reported pain scores. Clinically meaningful pain (≥3 on a 0–10 scale) at 30-days and 1-year after surgery were the primary and secondary outcomes, respectively. Of the study participants (N = 2106), 59% were female, 91% were non-Hispanic White, and the mean (SD) age was 62 (13) years; 41% of patients underwent orthopedic surgery and 88% received general anesthesia. Four acute pain trajectory clusters were identified. Pain trajectories were significantly associated with clinically meaningful pain at 30-days (*p* = 0.007), but not at 1-year (*p* = 0.79) after surgery using covariate-adjusted logistic regression models. Compared to Cluster 1, the other clusters had lower statistically significant odds of having pain at 30-days after surgery (Cluster 2: [OR = 0.67, 95%CI (0.51–0.89)]; Cluster 3:[OR = 0.74, 95%CI (0.56–0.99)]; Cluster 4:[OR = 0.46, 95%CI (0.26–0.82)], all *p*<0.05). Patients in Cluster 1 had the highest cumulative likelihood of pain and pain intensity during the latter half of their acute hospitalization period (48–96 hours), potentially contributing to the higher odds of pain during the 30-day postsurgical period. Early identification and management of high-risk pain trajectories can help in ascertaining appropriate pain management interventions. Such interventions can mitigate the occurrence of long-term disabilities associated with pain.

## Introduction

Nearly 300 million surgeries are performed worldwide every year [1]. Acute pain after surgery is common, and when untreated, leads to poor patient satisfaction, longer hospital length of stay, lower health-related quality of life, and non-compliance with rehabilitation regimens [2–6]. In some patients, postsurgical pain lingers after the injured tissues have healed; pain that persists for 3 months or longer after surgery is referred to as persistent postsurgical pain (PPSP) [7] and affects 5–35% of all surgical patients [8].

Recent research has highlighted that acute postsurgical pain is consistently associated with PPSP [9] and the continuation of the experience of pain [10–14], especially in the case of thoracotomy, breast, and knee surgeries [15–18]. However, acute pain is affected by the method of pain assessment [9], patient characteristics, pharmacotherapy decisions, type of surgery, presence of surgical complications, and the degree of tissue damage [19, 20]. One approach for contextualizing acute postsurgical pain is to represent it as a trajectory [21–23]. As opposed to a single measurement or the mean of a number of measurements, trajectories provide both a statistical and a visual approach to represent a patient’s experience of pain by highlighting pain characteristics such as the time of onset of pain, pain intensity, the rate of resolution or increase, and the consistency of pain relief [13, 19, 21].

Despite the potential promise of pain trajectories, much of the prior research has relied on characterizing the mean “shape” of pain trajectories [13, 22, 24, 25]. More sophisticated modeling approaches relying on group-based trajectory analysis have been utilized for characterizing the clinical characteristics associated with pain trajectories [14]. Researchers [5, 26] have also investigated the association between postsurgical pain trajectories and longer-term outcomes. However, these studies have relied on characterizing the mean pain trajectory, overlooking its more granular components such as the likelihood of pain or its intensity.

We have two exploratory research objectives. First, to investigate and classify postsurgical patients based on the longitudinal trends in their propensity for and intensity of acute pain during the postsurgical hospitalization period. Second, to assess the association of pain trajectory cluster membership with 30-day and 1-year pain using a retrospective study design.

## Method

### Study setting and participants

Data for this study were collected from adult patients (age ≥18) undergoing surgical procedures at Barnes-Jewish Hospital in St. Louis, Missouri, a tertiary academic medical center. Participants were recruited as part of the Systematic Assessment and Targeted Improvement of Services Following Yearlong Surgical Outcomes Surveys (SATISFY-SOS; NCT02032030) registry (the details of the registry, its design and development can be found here [27]). This longitudinal perioperative registry collected comprehensive preoperative, intraoperative, and postoperative data on clinical, physical, and mental health status of surgical patients.

**Fig 1:**
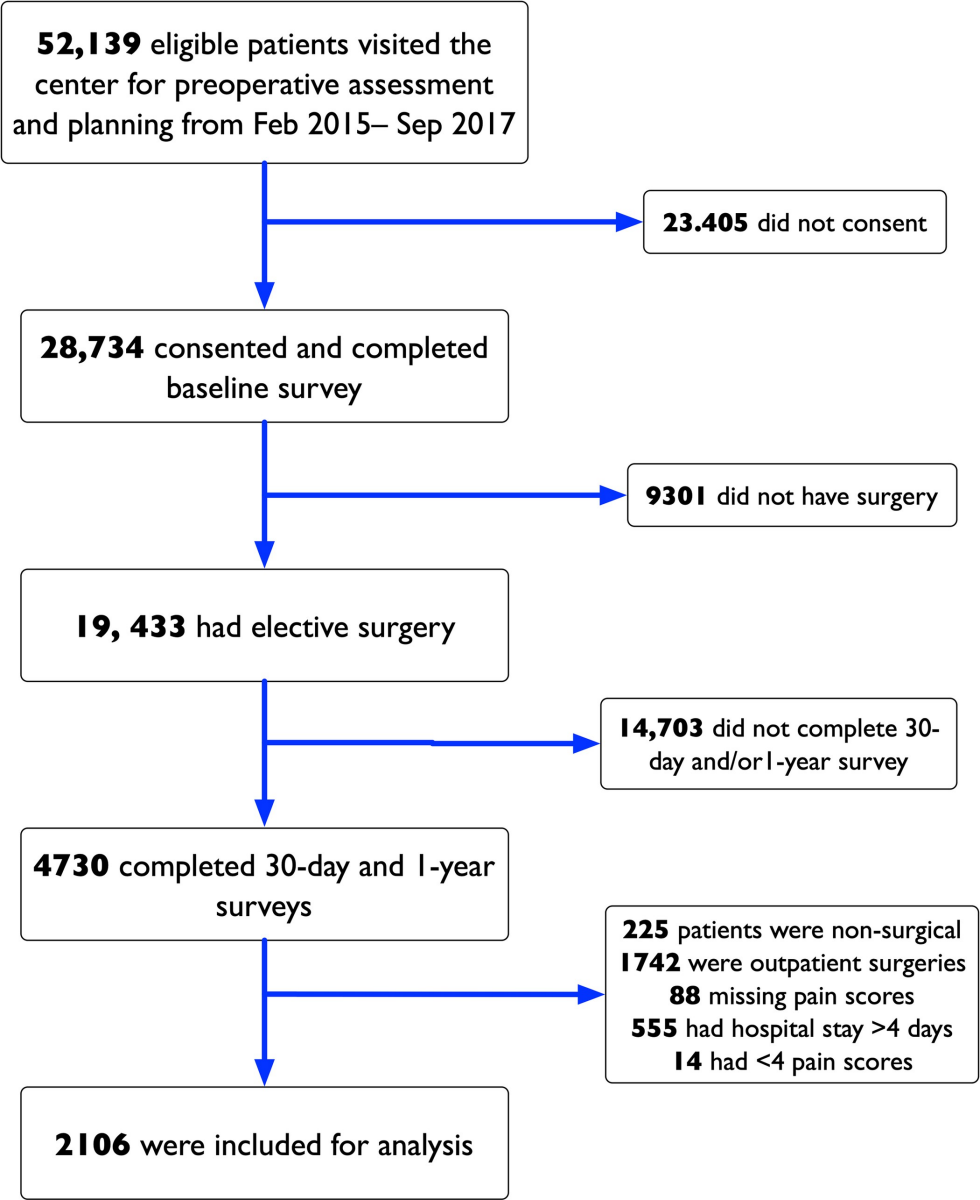
Patient selection for the study.

Patients were included in the SATISFY-SOS study if they were ≥18 years of age, English-speaking, able to provide consent, and did not have a diagnosis of dementia. Patients provided a written informed consent and agreed to be contacted at approximately 30-days and 1-year after surgery. As part of this study, we included all patients who gave consent between February 1, 2015 and September 30, 2017 (32 months), underwent invasive surgeries, had an inpatient stay lasting at least 24 hours, and completed follow up assessments at 30-days and 1-year after surgery. We excluded patients from the cohort if they had greater than 4-days of inpatient stay and/or fewer than 4 recorded pain scores during their inpatient stay. A summary of the patient selection and inclusion for this study is provided in Fig 1. The current study was approved with a waiver of consent by the institutional review board of Washington University (IRB#201901004). The reporting of this study followed the STrengthening the Reporting of OBservational Studies in Epidemiology (STROBE) guidelines for observational studies [28].

### Primary outcome and variables

The primary outcome was based on the patient response to the question on the 30-day post-surgery survey: “Currently, do you have any pain in your surgical incision or in the area related to your surgery,” (response = yes), followed by “on a scale of zero to ten, with zero being no pain and ten being the worst pain, what was your average pain level during the past week?” The self-reported pain intensity was based on a 0 to 10 numerical rating scale (NRS, where a pain of 0 represented no pain, and 10 represented worst pain). Based on the response to this question, we dichotomized patients as having clinically meaningful pain (≥3, threshold based on prior research [29, 30]) or not at 30-days after surgery, as the primary outcome. As a secondary outcome, we considered clinically meaningful pain at the surgical site at 1-year after surgery using the response to the same question on the 1-year survey.

From the patient’s electronic health record (EHR), we retrieved acute pain scores and time of the scores recorded during their inpatient stay, based on the 0–10 NRS as well. This sequence of pain scores was used for trajectory assessment and formulating trajectory classes, which constituted the primary exposure variable.

Based on the preoperative survey, we included the following variables: patient demographics (age, sex, and race), smoking status (0 = non-smoker, 1 = smoker, 2 = former smoker), patient acuity based on Charlson’s comorbidity index (CCI), surgery type, American Society of Anesthesiologists Physical Status (ASA-PS) classification (1–6), and anesthesia type (general, regional).

We also recorded the patient’s presurgical pain intensity at the surgical location. Surgery type was grouped into the following categories: minimally invasive surgery (MIS), breast surgery, cardiothoracic surgery, orthopedic surgery, vascular surgery, and other surgery. The “other” category included surgeries performed by colorectal, hepatobiliary, gynecological, ophthalmological, transplant, and gastroenterology services.

### Statistical analyses

Descriptive statistics of patients’ presurgical characteristics were first computed. Means and standard deviations were estimated for continuous variables; counts and percentages were obtained for categorical variables. Patients were then clustered into groups that shared a common pain trajectory based on their sequentially reported pain scores in the first 96 hours following surgery. These clusters were identified using the *traj* package [31, 32] in the R statistical programming package, which uses a 3-step machine learning approach starting with (a) 24 trajectory features that capture elements of linear and non-linear change, abrupt short-term fluctuations and deviations from monotonicity, and early versus late changes in pain score were first calculated for each patient (Section B in S1 File). Next, (b) principal component analysis (PCA) of these measures was conducted to account for intra-feature correlations. Lastly, (3) k-means clustering based on the resulting factors derived from the PCA factor loadings was used to group patients into latent trajectory groups. The appropriate number of clusters was determined using the cubic clustering criterion [33] implemented in the *NbClust* R package [34].

Bivariate associations between baseline characteristics and latent group membership (i.e., cluster), which was treated as a categorical variable where each level corresponded to a trajectory class identified by the *traj* method, were then analyzed. The significance of each association was ascertained using the Chi-square test of independence for categorical baseline variables and ANOVA F-test for continuous variables; the Kruskal-Wallis test was used when data were heavily skewed.

Finally, the relationships between pain trajectory latent groups and whether a patient experienced clinically meaningful pain at 30-days and 1-year after surgery were estimated. These relationships were modeled using a pair of logistic regression models, which adjust for baseline covariates. Backward selection was applied to obtain a parsimonious model of consequential variables.

Cluster-specific pain trajectories were modeled using zero-inflated Conway-Maxwell Poisson (ZICMP) mixed effects regression [35]. This method considered both linear and quadratic time as predictors and was built on the basis of a Conway-Maxwell Poisson distribution, which allows for over-dispersion in count data. The resulting model decomposes pain score trajectories into the likelihood of experiencing pain (i.e., the zero-inflation sub-model) and its mean intensity when experienced (i.e., the count sub-model). For each of the sub-trajectories the subject-level areas under the curve (AUC) [36] (i.e., area under the pain intensity-time curve and area under the probability of experiencing pain) relative to the maximum AUC at the end of the study period (i.e., 96 hours) was computed. Normalized AUCs were averaged by cluster at 8-hour intervals and plotted to illustrate cluster differences. Model parameters were estimated using maximum likelihood procedures implemented in the *glmmTMB* package in R [37].

Additional details of the statistical framework, latent class procedure, zero-inflated modeling, the assessment of model assumptions, and the predictive properties of the prognostic models of long-term pain are provided in the Sections A–H in S1 File.

## Results

### General characteristics

Of the overall analytic sample (N = 2106) of surgical patients, 59% were female, 91% were non-Hispanic White, and the mean (SD) age was 62 (13) years. Charlson Comorbidity Index (CCI) values were 0 (98% 10-year survival, 38% of our cohort), 1 (96% 10-year survival, 20%), and 2 (90% 10-year survival, 42%). The most common type of surgery was orthopedic (41%), and 88% of subjects received general anesthesia. Prior to surgery, 66% of patients reported minimal or no pain at the surgical site. Postoperatively, clinically meaningful pain in the surgical area was reported by 23% of the patients at 30 days and by 16% of patients 1-year after surgery. Rates of missing data were highest for age (3.1%), while the remaining covariates had a <1% missing data rate (Table 1).

**Table 1:** Baseline characteristics of study subjects by trajectory cluster.

	Overall (N = 2106)	Cluster1 (N = 542)	Cluster2 (N = 753)	Cluster3 (N = 696)	Cluster4 (N = 115)	p-value^a^
**Sex**						
Female	1237 (59%)	313 (58%)	431 (57%)	434 (62%)	59 (51%)	0.0629
Male	869 (41%)	229 (42%)	322 (43%)	262 (38%)	56 (49%)	
**Smoking Status**						
Smoker	1326 (63%)	339 (63%)	485 (64%)	425 (61%)	77 (67%)	0.252
Non-Smoker	153 (7%)	35 (6%)	46 (6%)	65 (9%)	7 (6%)	
Former Smoker	625 (30%)	168 (31%)	222 (29%)	204 (29%)	31 (27%)	
Missing	2 (0.1%)	0 (0%)	0 (0%)	2 (0.3%)	0 (0%)	
**Age (Years)**						
Mean (SD)	62 (± 13)	62 (± 13)	62 (± 13)	61 (± 13)	64 (± 12)	0.738
Missing	66 (3.1%)	16 (3.0%)	24 (3.2%)	23 (3.3%)	3 (2.6%)	
**Race**						
White	1922 (91%)	481 (89%)	698 (93%)	639 (92%)	104 (90%)	0.105
Black	149 (7%)	51 (9%)	44 (6%)	45 (6%)	9 (8%)	
Other	14 (1%)	7 (1%)	4 (1%)	3 (0%)	0 (0%)	
Missing	21 (1%)	3 (0.6%)	7 (0.9%)	9 (1.3%)	2 (1.7%)	
**Charlson Comorbidity Index**						
0 [98% 10-year Survival]	796 (38%)	231 (43%)	284 (3%)	234 (34%)	47 (41%)	**0.0077**
1 [96% 10-year Survival]	416 (20%)	110 (20%)	156 (2%)	127 (18%)	23 (20%)	
2 [90% 10-Year Survival]	894 (42%)	201 (37%)	313 (42%)	335 (48%)	45 (39%)	
**ASA** ^**d**^						
I	1170 (56%)	313 (58%)	443 (59%)	357 (51%)	57 (50%)	**0.0111**
Ill	936 (44%)	229 (42%)	310 (41%)	339 (49%)	58 (50%)	
**Surgical Category**						
Breast	58 (3%)	8 (1%)	23 (3%)	26 (4%)	1 (1%)	**< 0.0001**
Cardiothoracic	93 (4%)	16 (3%)	28 (4%)	44 (6%)	5 (4%)	
Minimally Invasive Surgery	134 (6%)	23 (4%)	57 (8%)	51 (7%)	3 (3%)	
Orthopedic	855 (41%)	355 (65%)	277 (37%)	169 (24%)	54 (47%)	
Other	891 (42%)	131 (24%)	339 (45%)	378 (54%)	43 (37%)	
Vascular	75 (4%)	9 (2%)	29 (4%)	28 (4%)	9 (8%)	
**Acute Pain**						
Mean (SE)^b^	3.05 (0.04)	2.72 (0.06)	2.94 (0.05)	3.87 (0.06)	0.38 (0.14)	<0.0001
**Baseline Pain Related to Surgery**						
Clinically meaningful^c^	712 (33.9)	196 (36.3)	247 (32.8)	229 (33.0)	40 (34.8)	0.562
Mean (SD)	2.5 (± 2.9)	2.6 (± 2.9)	2.5 (± 2.9)	2.5 (± 2.8)	2.4 (± 2.7)	0.267
**Pain at 30 days**						
Clinically meaningful	475 (22.6)	145 (26.8)	156 (20.7)	158 (22.7)	16 (13.9)	**0.008**
Mean (SD)	1.3 (± 2.1)	1.5 (± 2.3)	1.2 (± 2.1)	1.3 (± 2.2)	0.96 (± 1.8)	**0.0336**
**Pain at 1 year**						
Clinically meaningful	296 (14.1)	85 (15.7)	100 (13.3)	97 (13.9)	14 (12.2)	0.592
Mean (SD)	0.86 (1.93)	0.92 (1.95)	0.83 (1.91)	0.88 (1.97)	0.64 (1.63)	0.533
**Anesthesia Type**						
General	1844 (88%)	420 (77%)	680 (90%)	653 (94%)	91 (79%)	**<0.0001**
Regional	256 (12%)	121 (22%)	70 (9%)	41 (6%)	24 (21%)	
Missing	6 (0.3%)	1 (0.2%)	3 (0.4%)	2 (0.3%)	0 (0%)	

a: P-values were based on Chi-square and ANOVA F tests when samples were independent.

b: Means, standard errors (SE) and corresponding p-value were computed using a linear mixed effects model with random intercept to account for clustering within patient.

c: Clinically meaningful pain corresponds to an NRS pain score ≥ 3.

d: No patients had ASA II level.

### Acute pain trajectory clusters

**Fig 2:**
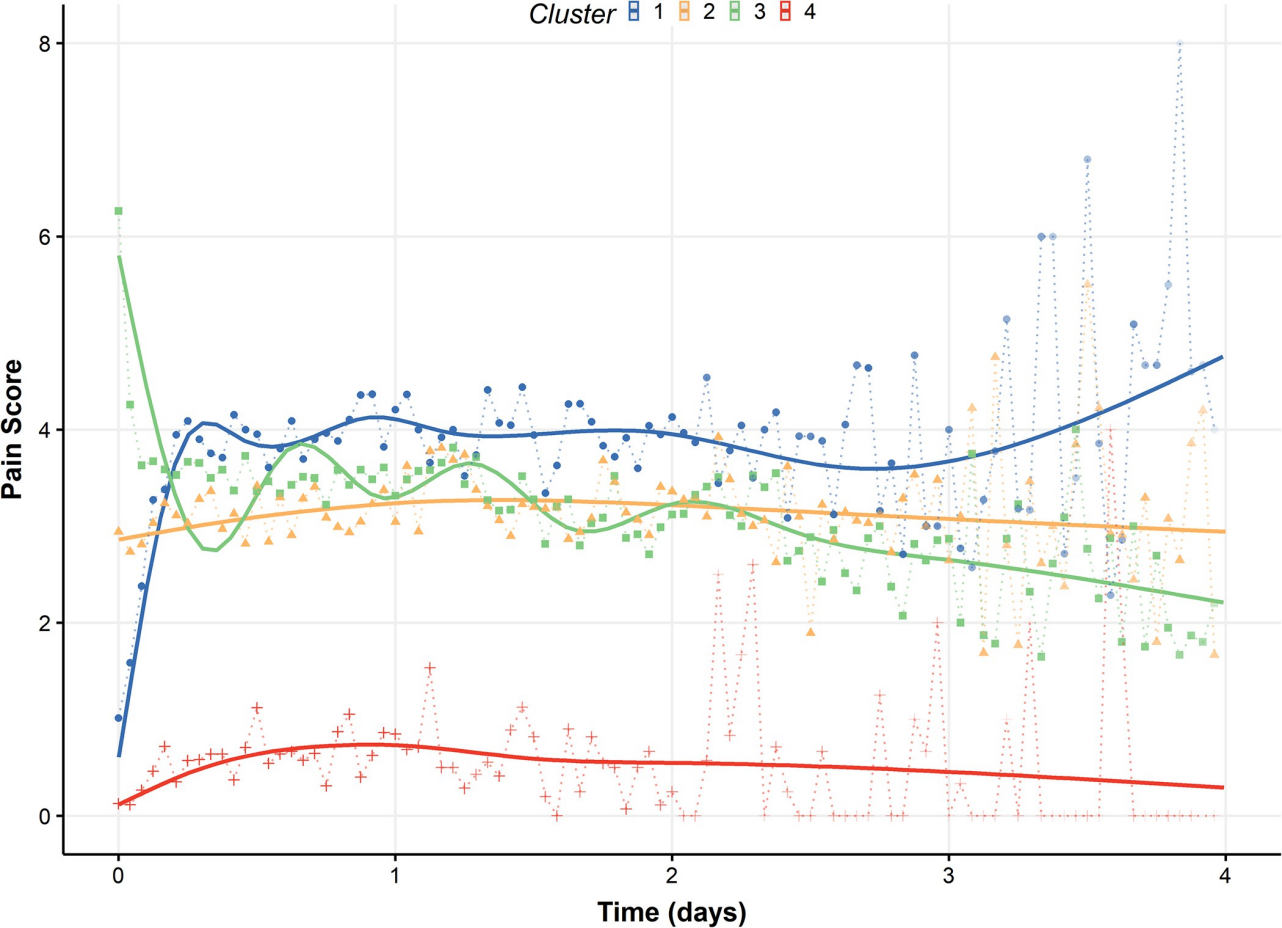
Estimated acute pain trajectories by cluster. The points and the dotted lines represent the mean acute pain of patients during each hour of observation. The solid lines show loess-smoothed pain trajectories. Areas under the curve (AUC) for each cluster (1–4) are 549095, 519615, 667908, and 877505, respectively.

**Fig 3:**
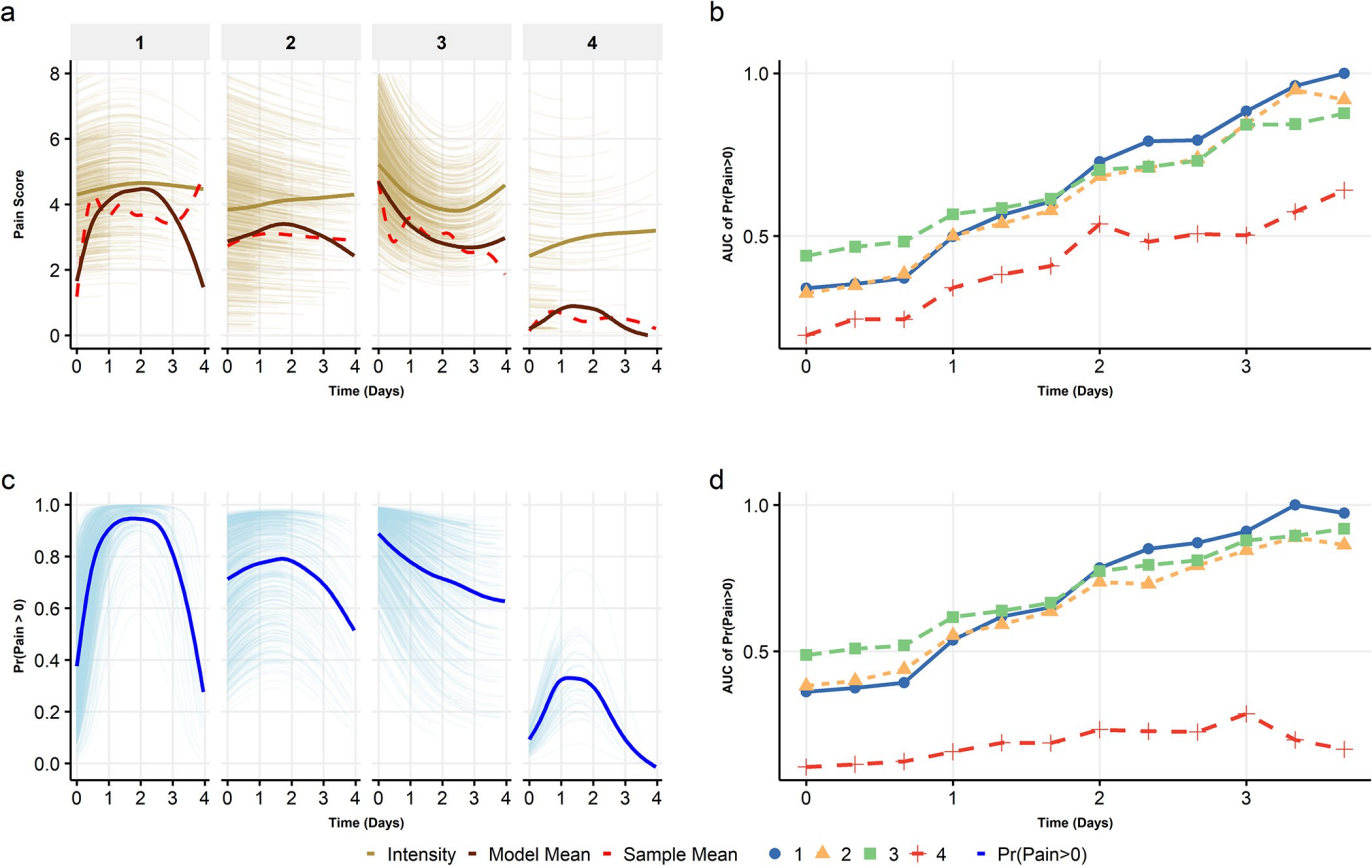
(a) shows the by cluster (1–4) sample mean pain (red dashed line), model predicted mean (solid brown), and conditional predicted pain (gold) over time. (b) gives the area under the curve (AUC) of pain intensity every six hours for each cluster. (c) is the model estimated mean likelihood of experiencing pain over time for each cluster, and (d) is the corresponding AUC for six-hour intervals. AUCs were normalized using the maximum AUC to obtain consistent scales.

We identified four pain trajectory clusters, comprised of 542, 753, 696, and 115 patients, respectively (Fig 2). Cluster 3 had the highest area under the pain intensity time curve (AUC = 667908) and highest mean experienced pain intensity (*M* = 3.87), followed by Cluster 1 (AUC = 549095, *M* = 2.72), Cluster 2 (AUC = 519615, *M* = 2.94), and Cluster 4 (AUC = 87705, *M* = 0.38). These summary values, however, do not reflect changes in trajectory trends during the postsurgical hospitalization period (Fig 3). An assessment of the clustering approach showed that the principal component scores provided reasonable discernment between groups with minimal overlap across clusters (Section C in S1 File).

Cluster membership was found to be significantly associated with CCI, ASA-PS, surgery type, anesthesia type, and mean pain at 30-days (Table 1). There were also marginal trends between cluster membership and patients’ sex and race. Neither presurgical pain nor pain at 1-year after surgery were significantly associated with acute pain trajectory clusters (Table 1).

Cluster 1 had the highest percentage of patients who received regional anesthesia (22%) and patients undergoing orthopedic surgeries (65%), as well as the highest average presurgical [*M* = 2.6 (SD = 2.9)], 30-day [1.5 (2.3)] and 1-year [0.9 (1.9)] pain. Patients in Cluster 2 predominantly received general anesthesia (90%) and underwent orthopedic (37%) or other (45%) surgeries. Cluster 3 included patients with the highest percentage of females (62%), received predominantly general anesthesia (94%), had the majority of surgeries in the other category (54%), and reported the highest intensity of initial postoperative acute pain (~6). Cluster 4 was similar to cluster 1 in terms of predominantly orthopedic surgeries (54%) and a high percentage of patients receiving regional anesthesia (21%), but it included patients with the least average acute postoperative pain (*M* = 0.38) and was associated with the lowest mean pain intensity at the surgical site at 30-days [0.9 (1.8)] and at 1-year after surgery [0.6 (1.6)].

### Effect of pain trajectories on 30-day and 1-year pain

The covariate-adjusted association between cluster membership was significant on clinically meaningful postsurgical pain at 30-days (p<0.01), but not at 1-year after surgery (p = 0.79) (Table 2). Cluster 1 membership was associated with increased odds of clinically meaningful lingering pain at 30-days. Cluster 4, which was characterized by low mean acute pain intensity and low prevalence of pain (i.e., only ~30% of the patients in this cluster had any postoperative pain; see Fig 3) and was associated with the lowest risk of lingering pain [OR = 0.46, 95%CI (0.26–0.82)]. The cluster associations (i.e., odds ratios) with 30-day pain were adjusted for age, race, sex, smoking, CCI, type of anesthesia, and presurgical pain; ORs for 1-year pain were adjusted for smoking, CCI, presurgical pain, and surgical category (Table 2; also, Table A3 in S1 File).

**Table 2:** Logistic regression models of 30-day and 1-year Moderate/Severe pain.

	30-Day Clinically Meaningful Pain			1-Year Clinically Meaningful Pain		
*Predictors*	*Odds Ratio* ^*b*^	*95% CI*	*p-value*	*Odds Ratio* ^*c*^	*95% CI*	*p-value*
(Intercept)	0.27	0.15–0.49	**<0.001**	0.58	0.23–1.46	0.250
Cluster^a^						
2	0.67	0.51–0.89	**0.005**	0.86	0.62–1.20	0.380
3	0.74	0.56–0.99	**0.041**	0.87	0.61–1.23	0.421
4	0.46	0.26–0.82	**0.009**	0.80	0.43–1.50	0.493

Notes: a: Type III likelihood ratio Chi-square tests of overall cluster effect yielded p-value = .007 at 30 days and p-value = .79 at 1 year. b: ORs adjusted for age, race, sex, smoking, CCI, anesthesia, and baseline pain. c: ORs adjusted for smoking, CCI, baseline pain, and surgical category.

Based on the Hosmer-Lemeshow goodness-of-fit test, both models of 30-day and 1-year pain fit the data well (p = 0.34 and 0.91 respectively); a receiver operating curve (ROC) assessment with re-sampling indicated that the associations were not sensitive to sample reduction (Section H in S1 File).

### Characteristics of acute pain trajectory clusters

The ZICMP mixed effects model (Fig 3) shows the decomposition of each cluster-specific mean trajectory into: (a) likelihood of experiencing pain (pain>0)––i.e., zero-inflation––over time (Fig 3C, blue line); and (b) mean intensity of experienced pain––i.e., conditioned on non-zero pain––reported at each time point (Fig 3A, gold line).

Cluster 1 was characterized by an inverse U-shaped likelihood of pain amongst the patients: 40% of patients initially experienced pain for 24 hours (Fig 3C), followed by a steady increase where ~80% of the patients experienced pain between 24 and 72 hours, and then a gradual decrease to 25% of the patients having pain (72–96 hours). The mean experienced pain was nearly homogenous over time with a pain score ranging from 4–4.5 (Fig 3A, gold line), highlighting that those patients who experienced pain did so consistently at a high level.

For Clusters 2 and 3, the pain likelihood patterns were relatively similar. The number of patients experiencing pain in Cluster 2 was initially high (~75% over first 48 hours), followed by a gradual decrease to ~50%. Similarly, a large percentage of patients (~85%) in Cluster 3 initially had pain; however, the likelihood of pain among patients in this cluster slowly decreased over time, to ~40% by day 4. In both these clusters, the mean experienced pain intensity was similar to Cluster 1 (~5, on a 0–10 NRS) over the hospitalization period. In contrast, Cluster 4 was characterized by the lowest likelihood of pain (≤ 25% of patients experienced pain for 24–48 hours). However, the reported pain intensity among those experiencing pain was higher than expected, initially at 3, on 0–10 scale, and increasing to ~4 by day 4.

In Fig 3A, the sample hourly mean pain scores (dotted red line) and the model predicted mean scores (solid brown line) align well, suggesting that the model provides a reasonable fit of the data (Tables A1, A2 in S1 File). These results suggest that the overall differences in trajectories were driven by the zero-inflated portion of the model; this is not unsurprising given that experienced pain intensity trajectories across clusters were relatively similar (Fig 3A), while the trajectories of the likelihood of zero pain were far more heterogeneous (Fig 3C).

We also computed and plotted the AUC for pain intensity (Fig 3B) and likelihood of pain (Fig 3D) for each of the clusters. For Cluster 1, pain intensity and likelihood of pain were lower than or similar to Clusters 2 and 3 during the initial ~48 hours after surgery. However, pain intensity and the likelihood of pain for Cluster 1 became the highest during the latter half of the hospitalization period (48–96 hours). This potentially points to a more intense pain experience during the later phase of hospitalization (and possibly, at discharge) that contributed to the highest odds of 30-day postsurgical pain among patients in this cluster, rather than the mean pain during the acute postsurgical period.

## Discussion

In this study, we modeled the acute postsurgical pain trajectories of patients and identified four clusters of postsurgical pain trajectories. The primary results of the study showed that acute pain trajectories were significantly associated with lingering pain reported at 30-days after surgery. From a methodological standpoint, we further examined the structural properties of these trajectories by using a novel approach, relying on the longitudinal zero-inflated Conway-Maxwell Poisson (ZICMP) models. This approach provided analytic mechanisms to jointly model, in a longitudinal manner, the likelihood of experiencing any pain, and the mean intensity of pain, when experiencing pain. Results of ZICMP modeling of pain trajectories by cluster highlighted relationship between the different types of pain experiences across time—periods of zero pain and periods of varying pain intensity—and lingering pain. In context, these findings suggest that trajectory analysis models that solely rely on mean pain over time, as is the case with most previous research [21, 26], may overlook more nuanced characteristics of trajectories and their associations with long-term outcomes, and thus, may paint an incomplete picture of a patient’s pain experience during the acute hospitalization period.

One critical structural nuance that, to our knowledge, has been overlooked in previous studies of pain trajectories is the vast discrepancy between mean pain and experienced pain intensity. The experienced pain intensities across all clusters were clinically high (>3 for all clusters; ~5 in Clusters 1 and 2), whereas the likelihood of pain was heterogeneous across the clusters. Although the patients in Cluster 3 experienced the highest mean intensity of acute pain (*M* = 3.87), the likelihood of pain at 30-days was highest for Cluster 1 where patients had the highest cumulative likelihood of pain and pain intensity during the latter half of their acute hospitalization period (48–96 hours; Fig 3B & 3D). Such effects were also evident in other clusters: in Cluster 4 the observed low average pain (red dotted line, Fig 3A) was primarily a manifestation of the fact that most patients did not report experiencing any pain; however, when pain was reported, its level was clinically meaningful (and high). Moreover, although Clusters 2 and 3 had substantial overlap in mean trajectory (Fig 2), both their zero-inflation (Fig 3C) and, to a lesser degree, their pain intensity trajectories differed over time.

There are several potential advantages of characterizing and identifying the experienced pain trajectories of postsurgical patients. First, appropriately classifying a patient into one of the experienced pain trajectory clusters can help in ascertaining their odds of having lingering pain at 30-days (or longer). Second, the identification of the nuances of pain trajectories is important for managing patients by developing targeted pain care interventions. Such interventions can include focused acute pain management during the perioperative period [38] and transitional pain care after hospital discharge [39]. For example, patients in Cluster 1, who are likely to have higher pain towards the end of their hospitalization, can benefit from pain management planning at hospital discharge. Such decisions can include outpatient referrals or pain management services for multi-modal analgesic management [26, 40]. As untreated pain can have a significant impact on the risk of opioid use and opioid use disorders, pharmacological and behavioral interventions should be tested to mitigate risk in such high risk groups [5].

This was an exploratory retrospective study with a relatively large sample of self-selected participants undergoing major surgery. The findings are preliminary and afford opportunities and directions for further research on assessing the role of postsurgical pain trajectories in patient care management. For translating acute pain trajectories to the point-of-care, several challenges exist. First, models should be developed about predicted patient pain trajectory at different time points during the surgical care continuum. Expected pain trajectory projections at various points in the surgical care continuum—after surgery and at multiple time points during the acute period (e.g., at 12-hour intervals)—can help in the proactive management of pain; additionally, real-time identification of a patient’s expected trajectory can help in the development of clinical decision support tools that can assist in decision making about pain and medication management.

Although our results indicate a strong association between trajectories and 30-day pain, personalized methods of trajectory characterization that do not rely on cluster membership are needed for clinical translation. One such potential approach is the use of joint modeling of the relationship between individual random effects exhibited in the longitudinal model and long-term pain [41]. To our knowledge, this approach is yet to be utilized in this context and our results suggest that joint models ought to incorporate both experienced pain and zero-inflation (likelihood of experiencing pain).

The clusters also showed discriminant validity, highlighting groups of patients expected to have different experiences of postsurgical pain. In other words, clusters represented groups of patients that had similar experiences of pain (and no pain), suggesting that the patients in these clusters could be distinguished based on surgical or clinical characteristics [21]. For example, patients in Cluster 1 had the highest percentage of regional anesthesia, with a large percentage of patients experiencing lower pain (as expected) during the early acute period.

Finally, from a methodological perspective, our approach, which exploits prior work in machine learning and statistical modeling, can identify and characterize non-polynomial trajectories without constraining *a priori* assumptions about the functional form of the trajectory and the distribution of class membership (as in parametric mixture models), and accommodates irregular observation intervals and variable observation periods. Furthermore, the machine learning method for clustering patients works by delineating trajectories into 24 functional characteristics ranging from univariate descriptors of location and scale, to linear and non-linear changes. This provides a critical balance between stationary pain readings, which are intrinsically subjective and highly variable, and relative pain scores over time, which underscore trends. As opposed to linear models [22] and mean trajectory clusters [26], our approach helped in ascertaining the experience of pain and zero pain within trajectory clusters, highlighting the complexities of meaningfully utilizing pain trajectories for acute pain management, while mitigating the impact of pain subjectivity.

This study has several limitations. Acute pain recordings were obtained from the patients’ electronic record. It is possible that there was irregularity in the timing of pain score recordings; however, our findings (Section G in S1 File) do not indicate that this is related to the level of experienced pain. Similarly, the timing of the recording of the pain scores in the electronic record may have been delayed by local clinical practices (e.g., nurses recording multiple pain scores for a patient at the end of their shift). The pain trajectories could also have been affected by the nature of surgery, presurgical pain, and anesthesia type (e.g., regional vs. general). However, it must be noted that the surgery type, presurgical pain and anesthetic type were adjusted for in the multivariable logistic regression.

As part of the SATISFY-SOS registry, we did not capture data on whether patients had pre-existing chronic pain at the surgical site or whether they were on specific pain management interventions. Baseline pain, which may serve as a reasonable proxy for historical experiences of pain, was incorporated in the logistic regression models used. The primary measurement of the 30-day and 1-year pain were obtained from self-reported surveys available from the SATISFY-SOS registry; these responses inherently included subjectivity in pain reports, and it is possible that these were affected by differential response rates. The lack of significant association between the pain trajectories and 1-year pain is potentially due to the lack of available mediating factors underlying the long-term recovery process, success and adherence to rehabilitation, other surgeries or related clinical events.

For all analyses, the recorded time of pain recording was used, which may not reflect the actual pain at that time. A cursory cross-validation study (results omitted) in which sub-samples of each patient’s data was used yielded trajectory clusters similar to those in Fig 2, suggesting that our approach is robust in the face of potential bias associated with the timing of the recording. Additionally, patients with higher pain and more complex surgeries may have been observed for longer periods of time. To account for these longer periods, we used a 4-day cutoff period for our analysis, which corresponds to a significant drop off in measurements. However, it is worth noting that this is one of few studies evaluating postsurgical pain trajectories utilizing acute in-hospital pain scores, which have higher granularity, but present new challenges for data processing, organization, and management.

We did not account for clinical interventions or medications (including opioids) that were provided during the acute period. This was primarily because of the lack of availability of comprehensive medication administration data from the EHR. At the time of the data collection, the intraoperative and acute patient care information was stored in separate electronic records and medication administration was tracked separately. Tracking accurate medication information (including that of opioids) was hence impossible. We acknowledge that postoperative opioid management strategies, which we did not utilize for this analysis, could have played a significant role in the evolution of pain trajectories. Similarly, we did not differentiate between various regional anesthesia sub-types; we dichotomized surgeries based on general anesthesia only versus anesthetic approaches that used regional anesthesia, either alone or in combination with general anesthesia. For example, under “regional anesthesia” we included approaches such as peripheral nerve blocks, epidural blocks with continuous catheter-based infusion, combined spinal epidural anesthesia, and single-shot spinal (intrathecal) blocks. We also note that the racial distribution of the study population (91% White) impedes the generalizability of our findings and hinders our ability to examine racial disparities in acute and chronic pain experiences [42].

## Supporting information

S1 File In this document we provide additional details on the following: A) Statistical framework of pain score analysis; B) latent trajectories of acute pain; C) assessment of the latent trajectory class method; D) properties of acute pain trajectories; E) effect of latent pain trajectories on 30-day and 1-year postsurgical pain; F) evaluation of logistic regression models; G) further evaluation of pain recording frequency; H) assessment of clustering and prognostic models using resampling.
(DOCX)

## References

[1] Nepogodiev D., Martin J., Biccard B., Makupe A., Bhangu A., and Ademuyiwa A., Global burden of postoperative death. Lancet, 2019. 393(401): p. 33139–8. doi: 10.1016/S0140-6736(18)33139-8 30722955

[2] Guimarães‐Pereira L., Farinha F., Azevedo L., Abelha F., and Castro‐Lopes J., Persistent postoperative pain after cardiac surgery: incidence, characterization, associated factors and its impact in quality of life. European Journal of Pain, 2016. 20(9): p. 1433–1442. doi: 10.1002/ejp.866 26988335

[3] Peters C.L., Shirley B., and Erickson J., The effect of a new multimodal perioperative anesthetic regimen on postoperative pain, side effects, rehabilitation, and length of hospital stay after total joint arthroplasty. The Journal of arthroplasty, 2006. 21(6): p. 132–138. 16950075 10.1016/j.arth.2006.04.017

[4] Wu C.L., Naqibuddin M., Rowlingson A.J., Lietman S.A., Jermyn R.M., and Fleisher L.A., The effect of pain on health-related quality of life in the immediate postoperative period. Anesthesia & Analgesia, 2003. 97(4): p. 1078–1085. doi: 10.1213/01.ANE.0000081722.09164.D5 14500161

[5] Singh J.A., Lemay C.A., Nobel L., Yang W., Weissman N., Saag K.G., et al., Association of Early Postoperative Pain Trajectories With Longer-term Pain Outcome After Primary Total Knee Arthroplasty. JAMA network open, 2019. 2(11): p. e1915105–e1915105. doi: 10.1001/jamanetworkopen.2019.15105 31722026 PMC6902788

[6] Rosenberg J. and Kehlet H., Does effective postoperative pain management influence surgical morbidity? European surgical research, 1999. 31(2): p. 133–137. doi: 10.1159/000008631 10213851

[7] Schug S.A., Lavand’homme P., Barke A., Korwisi B., Rief W., and Treede R.-D., The IASP classification of chronic pain for ICD-11: chronic postsurgical or posttraumatic pain. Pain, 2019. 160(1): p. 45–52. doi: 10.1097/j.pain.0000000000001413 30586070

[8] Haroutiunian S., Nikolajsen L., Finnerup N.B., and Jensen T.S., The neuropathic component in persistent postsurgical pain: a systematic literature review. PAIN®, 2013. 154(1): p. 95–102. doi: 10.1016/j.pain.2012.09.010 23273105

[9] Gilron I., Vandenkerkhof E., Katz J., Kehlet H., and Carley M., Evaluating the association between acute and chronic pain after surgery. The Clinical Journal of Pain, 2017. 33(7): p. 588–594. doi: 10.1097/AJP.0000000000000443 28145910

[10] Katz J., One man’s risk factor is another man’s outcome: difference in risk factor profiles for chronic postsurgical pain maintenance vs transition. Pain, 2012. 153(3): p. 505–506. doi: 10.1016/j.pain.2011.10.044 22100359

[11] Katz J., Jackson M., Kavanagh B.P., and Sandler A.N., Acute pain after thoracic surgery predicts long-term post-thoracotomy pain. The Clinical journal of pain, 1996. 12(1): p. 50–55. doi: 10.1097/00002508-199603000-00009 8722735

[12] Katz J. and Seltzer Z.e., Transition from acute to chronic postsurgical pain: risk factors and protective factors. Expert review of neurotherapeutics, 2009. 9(5): p. 723–744. doi: 10.1586/ern.09.20 19402781

[13] Althaus A., Arránz Becker O., and Neugebauer E., Distinguishing between pain intensity and pain resolution: Using acute post‐surgical pain trajectories to predict chronic post‐surgical pain. European Journal of Pain, 2014. 18(4): p. 513–521. doi: 10.1002/j.1532-2149.2013.00385.x 23983024

[14] Pagé M.G., Katz J., Escobar E.M.R., Lutzky-Cohen N., Curtis K., Fuss S., et al., Distinguishing problematic from nonproblematic postsurgical pain: a pain trajectory analysis after total knee arthroplasty. Pain, 2015. 156(3): p. 460–468. doi: 10.1097/01.j.pain.0000460327.10515.2d 25599235

[15] Gottschalk A. and Ochroch E.A., Clinical and demographic characteristics of patients with chronic pain after major thoracotomy. Clin J Pain, 2008. 24(8): p. 708–16. doi: 10.1097/AJP.0b013e318174badd 18806536

[16] Poleshuck E.L., Katz J., Andrus C.H., Hogan L.A., Jung B.F., Kulick D.I., et al., Risk factors for chronic pain following breast cancer surgery: a prospective study. J Pain, 2006. 7(9): p. 626–634. doi: 10.1016/j.jpain.2006.02.007 16942948 PMC6983301

[17] Puolakka P.A., Rorarius M.G., Roviola M., Puolakka T.J., Nordhausen K., and Lindgren L., Persistent pain following knee arthroplasty. European Journal of Anaesthesiology (EJA), 2010. 27(5): p. 455–460. doi: 10.1097/EJA.0b013e328335b31c 20299989

[18] Wang L., Guyatt G.H., Kennedy S.A., Romerosa B., Kwon H.Y., Kaushal A., et al., Predictors of persistent pain after breast cancer surgery: a systematic review and meta-analysis of observational studies. CMAJ, 2016. 188(14): p. E352–E361. doi: 10.1503/cmaj.151276 27402075 PMC5047835

[19] Okamoto A., Yamasaki M., Yokota I., Mori M., Matsuda M., Yamaguchi Y., et al., Classification of acute pain trajectory after breast cancer surgery identifies patients at risk for persistent pain: a prospective observational study. Journal of pain research, 2018. 11: p. 2197. doi: 10.2147/JPR.S171680 30323654 PMC6179582

[20] Willingham M., Rangrass G., Curcuru C., Abdallah A.B., Wildes T.S., McKinnon S., et al., Association between postoperative complications and lingering post-surgical pain: an observational cohort study. British journal of anaesthesia, 2020. 124(2): p. 214–221. doi: 10.1016/j.bja.2019.10.012 31771788

[21] Kannampallil T., Galanter W.L., Falck S., Gaunt M.J., Gibbons R.D., McNutt R., et al., Characterizing the pain score trajectories of hospitalized adult medical and surgical patients: a retrospective cohort study. Pain, 2016. 157(12): p. 2739. doi: 10.1097/j.pain.0000000000000693 27548045 PMC5113285

[22] Chapman C.R., Donaldson G.W., Davis J.J., and Bradshaw D.H., Improving individual measurement of postoperative pain: the pain trajectory. The Journal of Pain, 2011. 12(2): p. 257–262. doi: 10.1016/j.jpain.2010.08.005 21237721 PMC3052945

[23] Tighe P.J., The time course of acute pain in hospitalized patients: Exciting progress in data and methods. Pain, 2016. 157(12): p. 2623. doi: 10.1097/j.pain.0000000000000714 27682211 PMC5224868

[24] Chapman C.R., Fosnocht D., and Donaldson G.W., Resolution of acute pain following discharge from the emergency department: the acute pain trajectory. The Journal of Pain, 2012. 13(3): p. 235–241. doi: 10.1016/j.jpain.2011.11.007 22285610 PMC3294147

[25] Chapman C.R., Zaslansky R., Donaldson G.W., and Shinfeld A., Postoperative pain trajectories in cardiac surgery patients. Pain research and treatment, 2012. doi: 10.1155/2012/608359 22448322 PMC3289864

[26] Hah J.M., Cramer E., Hilmoe H., Schmidt P., McCue R., Trafton J., et al., Factors associated with acute pain estimation, postoperative pain resolution, opioid cessation, and recovery: secondary analysis of a randomized clinical trial. JAMA network open, 2019. 2(3): p. e190168–e190168. doi: 10.1001/jamanetworkopen.2019.0168 30821824 PMC6484627

[27] Helsten D.L., Abdallah A.B., Avidan M.S., Wildes T.S., Winter A., McKinnon S., et al., Methodologic considerations for collecting patient-reported outcomes from unselected surgical patients. Anesthesiology: The Journal of the American Society of Anesthesiologists, 2016. 125(3): p. 495–504. doi: 10.1097/ALN.0000000000001217 27355128

[28] Von Elm E., Altman D.G., Egger M., Pocock S.J., Gøtzsche P.C., and Vandenbroucke J.P., The Strengthening the Reporting of Observational Studies in Epidemiology (STROBE) statement: guidelines for reporting observational studies. BMJ, 2007. 85: p. 867–872. 10.2471/BLT.07.045120 PMC2636253 18038077

[29] Vila M.R., Todorovic M.S., Tang C., Fisher M., Steinberg A., Field B., et al., Cognitive flexibility and persistent post-surgical pain: the FLEXCAPP prospective observational study. British journal of anaesthesia, 2020. 124(5): p. 614–622. doi: 10.1016/j.bja.2020.02.002 32169255

[30] Attal N., Masselin-Dubois A., Martinez V., Jayr C., Albi A., Fermanian J., et al., Does cognitive functioning predict chronic pain? Results from a prospective surgical cohort. Brain, 2014. 137(3): p. 904–917. doi: 10.1093/brain/awt354 24441173

[31] Sylvestre M.-P., McCusker J., Cole M., Regeasse A., Belzile E., and Abrahamowicz M., Classification of patterns of delirium severity scores over time in an elderly population. International Psychogeriatrics, 2006. 18(4): p. 667. doi: 10.1017/S1041610206003334 16640798

[32] Leffondré K., Abrahamowicz M., Regeasse A., Hawker G.A., Badley E.M., McCusker J., et al., Statistical measures were proposed for identifying longitudinal patterns of change in quantitative health indicators. Journal of clinical epidemiology, 2004. 57(10): p. 1049–1062. doi: 10.1016/j.jclinepi.2004.02.012 15528056

[33] Sarle W.S., Cubic clustering criterion. 1983: SAS institute.

[34] Charad M., Ghazzali N., Boiteau V., and Niknafs A., NbClust: an R package for determining the relevant number of clusters in a dataset. Journal of Statistical Software, 2014. 61: p. 1–36.

[35] Sellers K.F. and Shmueli G., A flexible regression model for count data. The Annals of Applied Statistics, 2010: p. 943–961.

[36] Tuck E., A simple" Filon-trapezoidal" rule. Mathematics of Computation, 1967. 21(98): p. 239–241.

[37] Brooks M.E., Kristensen K., van Benthem K.J., Magnusson A., Berg C.W., Nielsen A.,. et al., glmmTMB balances speed and flexibility among packages for zero-inflated generalized linear mixed modeling. The R journal, 2017. 9(2): p. 378–400.

[38] American Society of Anesthesiologists Task Force on Acute Pain Management, Practice guidelines for acute pain management in the perioperative setting: an updated report by the American Society of Anesthesiologists Task Force on Acute Pain Management. Anesthesiology, 2012. 116(2): p. 248–273. doi: 10.1097/ALN.0b013e31823c1030 22227789

[39] Katz J., Weinrib A., Fashler S.R., Katznelzon R., Shah B.R., Ladak S.S., et al., The Toronto General Hospital Transitional Pain Service: development and implementation of a multidisciplinary program to prevent chronic postsurgical pain. Journal of pain research, 2015. 8: p. 695. doi: 10.2147/JPR.S91924 26508886 PMC4610888

[40] Tiippana E., Hamunen K., Heiskanen T., Nieminen T., Kalso E., and Kontinen V.K., New approach for treatment of prolonged postoperative pain: APS Out-Patient Clinic. Scandinavian Journal of Pain, 2016. 12(1): p. 19–24. doi: 10.1016/j.sjpain.2016.02.008 28850486

[41] Tsiatis A.A. and Davidian M., Joint modeling of longitudinal and time-to-event data: an overview. Statistica Sinica, 2004: p. 809–834.

[42] Anderson K.O., Green C.R., and Payne R., Racial and ethnic disparities in pain: causes and consequences of unequal care. The Journal of Pain, 2009. 10(12): p. 1187–1204. doi: 10.1016/j.jpain.2009.10.002 19944378

