# Supplementary material for: Effect of acute postsurgical pain trajectories on 30-day and 1-year pain
Source: PLoS One. 2022 Jun 10;17(6):e0269455. doi: 10.1371/journal.pone.0269455 (PMC9187125; doi:10.1371/journal.pone.0269455)
Supplement: S1 File — In this document we provide additional details on the following: A) Statistical framework of pain score analysis; B) latent trajectories of acute pain; C) assessment of the latent trajectory class method; D) properties of acute pain trajectories; E) effect of latent pain trajectories on 30-day and 1-year postsurgical pain; F) evaluation of logistic regression models; G) further evaluation of pain recording frequency; H) assessment of clustering and prognostic models using resampling. (DOCX) [file pone.0269455.s001.docx]

Supplementary Material

In this supplementary section, we detail the statistical procedures used in the paper, including the mathematical framework of the data and its analytic models. We also provide complete results of the data modeling that were not incorporated in the main article. Furthermore, we provide the results of assessing the appropriateness and performance of the methods through a series of diagnostic tests.

# Statistical framework of pain score analysis

A formal mathematical representation is provided to operationalize the statistical approaches used for this study. For subject $i=1\ldots N$, where $N$ is the sample size, let $Y_{i}\left( T \right)=Y{}_{i}$ be the pain score observed at a distal time $T$ (30 days post-surgery). Pain during hospitalization or acute pain was denoted for each subject by the vector $\boldsymbol{X}_{i}=\left( X_{i}\left( t_{i1} \right),\ldots,X_{i}\left( t_{im_{i}} \right) \right)^{T}$, where $t_{i}=\left( t_{i1}, \ldots,t_{im_{i}} \right)^{T}$ is the vector of observation times of pain during hospitalization from the recorded anesthesia stop time (used as a proxy for end of surgery), $t_{i0},$such that $t_{ik}=t_{ik}^{*}-t_{i0}$, where $t_{ik}^{*}$ is the recorded time, and $t_{i1}<t_{i2}<\ldots<t_{im_{i}}<T$; this anchors the time variable to a clinical origin and accommodates irregular observation times as well as a variable number and duration of observations, $m_{i}$, for each subject $i$. For brevity, we will henceforth use $\boldsymbol{X}_{i}=\left( x_{i1}, x_{i2},\ldots,x_{im_{i}} \right)^{T}$where $x_{ij}=X_{i}(t_{ij})$.

Covariates such as baseline demographic variables, type of surgery and anesthesia type were denoted by the $q$-dimensional vector $\boldsymbol{Z}_{\boldsymbol{i}}\boldsymbol{=}\left( Z_{i1},\ldots,Z_{iq} \right)^{T}$. The covariates were assumed to be exogenous or external to the model for analytical purposes (this assumption is revisited in the Discussion section); covariates can include main effects, non-linear transformations, and interactions. Hence, the observed data for subject $i$ is the set $\boldsymbol{\omega}_{\boldsymbol{i}}=\{Y_{\boldsymbol{i}},\boldsymbol{X}_{\boldsymbol{i}},{\boldsymbol{Z}_{\boldsymbol{i}}\boldsymbol{, t}}_{\boldsymbol{i}}\}$.

# Latent trajectories of acute pain

Pain trajectories were estimated for the acute post-surgical period beginning from the recorded anesthesia stop time for each subject until up to four days of inpatient stay ($t_{i0}<t_{im_{i}}\leq4 days \forall i$), where $m_{i}\geq4$, that is, each subject had at minimum four recorded pain readings.

The group-based pain trajectories and group classifications (latent classes) were estimated using a machine learning procedure implemented in the *traj* package in the R statistical language [1-3]. This approach does not require *a priori* specifications of the functional form of the trajectories (e.g., linear, quadratic) and can accommodate irregular longitudinal patterns. The latent classes or clusters were identified in three steps:

1. For each patient, a set of 24 features were computed based on their individual sequence of pain scores in the observation period (0-96 hours). These features summarize different domains of the pain trajectory:
   1. **Elementary measures of change** include the range, mean-over-time, standard variation, coefficient of variation, overall change (last minus first observation), mean change per unit time, change relative to first score, change relative to mean-over-time, the slope and R-squared of a linear regression model of pain score versus time.
   2. **Measures of non-linearity and inconsistency of change** such as maximum and standard deviation of consecutive scores ($\Delta_{\mathrm{ij}} = x_{i,j+1} - x_{\mathrm{ij}}$), the sd of a normalized per-unit-time change, the mean and maximum of absolute consecutive change, the ratio of absolute consecutive change to mean-over-time and the slope of the linear model of the trajectory, and the standard deviation of the ratio of consecutive change to the slope.
   3. **Measures of deviations from monotonicity and abrupt short-term fluctuations** that are based on second differences, that is, the difference in difference of consecutive scores; these include mean of second differences and their absolute values, max of absolute values, and the ratios of max absolute second differences to mean-over-time and mean absolute first difference of consecutive values; and, the ratio of mean absolute second differences to mean first differences.
   4. **Measures contrasting early and later change** including the ratio of early to later change, the ratio relative to early-to-total change, and the ratio of late-to-total change. Here, early and late changes are defined on the basis of whether the time occurs in the first or second half of observation based on median time of each subject.
2. In order to account for the potential of correlation between the features, factor analysis was conducted using the eigenvalues of the correlation matrix subjected to varimax rotation. Therefore, each subject was assigned a score based on a linear combination of their individual features, weighted on the basis of factor loadings. Five principal components (PC) or factors were used.
3. Lastly, cluster analysis based on k-means was employed to identify patients with similar factor values. In this application, we used the cubic clustering criterion to determine an optimal number of clusters. However, we do note (as have [1]), that there is no consensus on the best data driven approach to establish an optimal number of clusters.

# Assessment of the latent trajectory class method


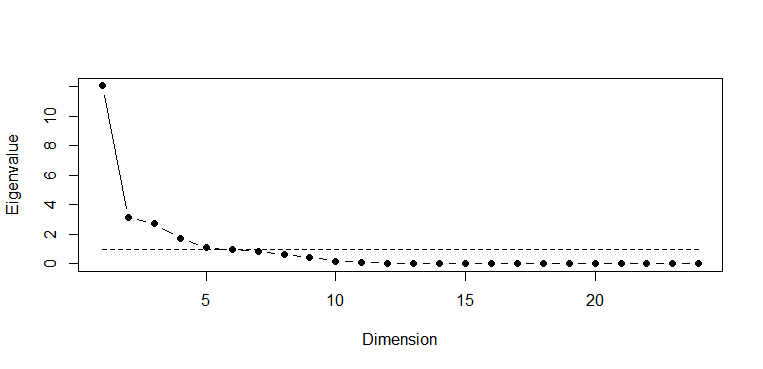


**Fig A1. Scree Plot Showing the Level of Variability Explained by Increasing Dimensions of the Principal Components Derived in Step 2 of the traj Procedure.**

The performance of the method was evaluated for steps 2 and 3 using standard machine learning diagnostics. To evaluate step 2 of the *traj* procedure, we used a Scree plot of the varimax correlation eigenvalues to ascertain whether the 5 selected PC’s corresponded to a reasonable level of explained variability in the 24 features of step 1. Based on the widely used “elbow” method [4] applied to the Scree plot in Fig A1, this appears to be a reasonable choice of PC’s.

To evaluate the cluster memberships identified in step 3, we used a three-dimensional plot of the correspondence between clusters and the first 3 PCs. Here, successful clustering would yield clearly distinct and well-separated regions occupied by each cluster in the three-dimensional space.

**
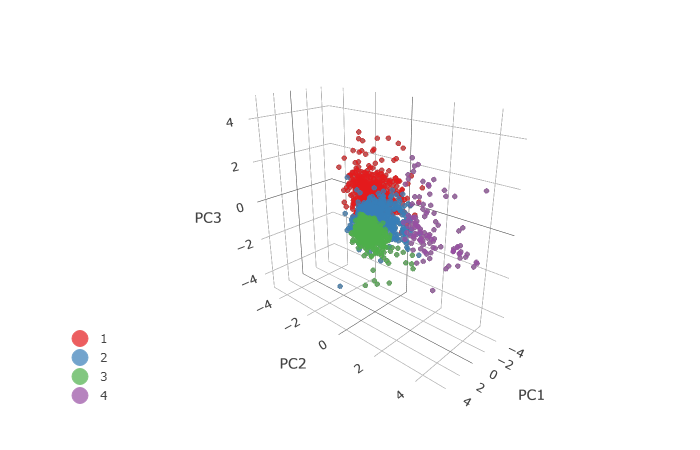
**

**Fig A2. A Three-Dimension Plot of the First Three Principal Component (PC's) Scores Produced by the traj Method.** The four trajectory clusters identified in step 3 are denoted by different colors. The figure was trimmed to remove outliers and improve visibility.

We can see from the results in Fig A2 that the first three PC’s yield reasonable discernment of the clusters. This is encouraging because the actual cluster memberships were identified on the basis of two additional PC’s, which of course would be difficult to display graphically in five dimensions.

# Properties of acute pain trajectories

Descriptive statistics of all baseline variables were calculated for each cluster and the significance of the relationships between baseline variables and clusters were assessed using Chi-square and ANOVA for categorical and continuous baseline variables, respectively.

As described in the Introduction of the main article, standard growth curve approaches focus almost entirely on mean or median trajectories over time, which can fail to capture the zero-inflation of pain scores. In this portion of the analyses, we used a time-dependent zero-inflated model in order to make a distinction between two simultaneously occurring components of pain trajectories: whether an individual experiences any pain at a particular time point and, if they do report any pain, the intensity of that experienced pain. In order to systematically explore these nuances of pain trajectories in each of the clusters, we modeled acute pain using mixed effects zero-inflated Conway-Maxwell Poisson (ZICMP) regression [5]. This approach extends the widely used ZI Poisson model [6] by accommodating the possibility of empirical over-dispersion. This approach assumes that the observed pain of each subject $i$ at time $t_{ij}$ is a mixture distribution of the form:

$$\boldsymbol{X}_{i}=\left\{ \begin{aligned} 0, & with probability \mathbf{p}_{i} \\ CMP(\boldsymbol{\lambda}_{i}), & with probability 1-\mathbf{p}_{i}, \end{aligned} \right.$$

where $\boldsymbol{p}_{i}=\left( p_{i1},\ldots,p_{im} \right)^{T}$ the time-specific probabilities of experiencing pain and $\boldsymbol{\lambda}_{i}=\left( \lambda_{i1},\ldots,\lambda_{im} \right)^{T}$ are and the mean parameters of a Conway-Maxwell Poisson used to model experienced pain for each subject. To account for within subject correlations, we incorporated random intercepts when estimating the model parameters using the canonical links $\log\left( \boldsymbol{\lambda}_{\boldsymbol{i}} \right)=\boldsymbol{Z}_{\boldsymbol{i}}^{T}\boldsymbol{\beta}+\nu_{\lambda i}$ and $\mathrm{logit} \left( \boldsymbol{p}_{\boldsymbol{i}} \right)=\boldsymbol{Z}_{\boldsymbol{i}}^{T}\boldsymbol{\alpha}+\nu_{pi}$. Here, $\nu_{\lambda i}$ and $\nu_{pi}$, the random intercept effects, are assumed to be bivariate normal; $\boldsymbol{Z}_{i}$ is a design matrix encompassing the model intercept, marginal effects of cluster membership, linear and quadratic time, and the interaction between cluster membership and both linear and quadratic time terms. The model was further modified to account for empirical limitations: first, the polynomial time function was *orthogonalized* to remove collinearity of linear and quadratic terms; second, pain scores were normalized using individual maximum pain as an offset in order to temper large trajectory fluctuations in patients with higher pain scores. Maximum likelihood methods in the R package *glmmTMB* [7] were used to estimate all parameters jointly. Estimates of the model parameters, their 95% confidence intervals, and p-values are given in Table A.1.

**Table A1. Maximum Likelihood Estimates, 95% Confidence Intervals and p-values of Mixed Effects ZIMCP Model Parameters.**

|  | **Zero-Inflated Model of Pain Trajectories** | | |  |
| --- | --- | --- | --- | --- |
| **Predictors** | **Estimates** | **95% CI** | **p-value** |  |
| **Count Model** | | | | |
| (Intercept) | -5.47 | -5.60 – -5.34 | **<0.001** |  |
| Time | 1.66 | -0.90 – 4.21 | 0.203 |  |
| Time^2 | -4.25 | -6.51 – -1.98 | **<0.001** |  |
| cluster [2] | 0.46 | 0.29 – 0.64 | **<0.001** |  |
| cluster [3] | -0.82 | -1.00 – -0.64 | **<0.001** |  |
| cluster [4] | 1.97 | 1.63 – 2.31 | **<0.001** |  |
| Time*cluster [2] | -9.33 | -12.55 – -6.11 | **<0.001** |  |
| Time^2*cluster [2] | 4.81 | 1.91 – 7.70 | **0.001** |  |
| Time*cluster [3] | -28.65 | -31.64 – -25.66 | **<0.001** |  |
| Time^2*cluster [3] | 16.87 | 14.15 – 19.58 | **<0.001** |  |
| Time*cluster [4] | -1.81 | -23.68 – 20.06 | 0.871 |  |
| Time^2*cluster [4] | 8.25 | -10.85 – 27.36 | 0.397 |  |
| **Zero-Inflated Model** | | | |  |
| (Intercept) | -1.05 | -1.18 – -0.92 | **<0.001** |  |
| Time | -249.27 | -267.06 – -231.48 | **<0.001** |  |
| Time^2 | 213.79 | 199.26 – 228.32 | **<0.001** |  |
| cluster [2] | -0.45 | -0.62 – -0.27 | **<0.001** |  |
| cluster [3] | -0.97 | -1.15 – -0.79 | **<0.001** |  |
| cluster [4] | 2.78 | 2.44 – 3.11 | **<0.001** |  |
| Time*cluster [2] | 236.73 | 215.79 – 257.67 | **<0.001** |  |
| Time^2*cluster [2] | -173.33 | -191.01 – -155.66 | **<0.001** |  |
| Time * cluster [3] | 369.60 | 348.93 – 390.26 | **<0.001** |  |
| Time^2*cluster [3] | -238.30 | -255.29 – -221.31 | **<0.001** |  |
| Time*cluster [4] | 184.38 | 137.04 – 231.71 | **<0.001** |  |
| Time^2*cluster [4] | -61.90 | -105.71 – -18.09 | **0.006** |  |

The ZICMP model in Table A1 (henceforth the full model) was compared to smaller nested models using the sample corrected Akaike Information Criterion (AICc). The results shown in Table A2 show that the full model significantly improves fit compared to all other nested models. Moreover, the effects of linear and quadratic time and their interactions with cluster membership was more substantial in the zero-inflated sub-model compared to the count sub-model (Model 4 and 5 vs Model 2 and 3). However, adding time to the count sub-model resulted in a significant improvement compared to Model 1.

**Table A2. Model Fit Characteristics.**

| *Models* | *Count Model* | *ZI Model* | *K* | *AICc* | *Delta AICc* | *LL* |
| --- | --- | --- | --- | --- | --- | --- |
| Full Model | $C\times(t+t^{2})$ | $C\times(t+t^{2})$ | 27 | 153243.25 | 0.00 | -76594.61 |
| Model 5 | $C+(t+t^{2})$ | $C\times(t+t^{2})$ | 21 | 153852.94 | 609.69 | -76905.46 |
| Model 4 | $C$ | $C\times(t+t^{2})$ | 19 | 154747.32 | 1504.06 | -77354.65 |
| Model 3 | $C\times(t+t^{2})$ | $C+(t+t^{2})$ | 21 | 155743.06 | 2499.80 | -77850.52 |
| Model 2 | $C\times(t+t^{2})$ | $C$ | 19 | 156333.02 | 3089.77 | -78147.50 |
| Model 1 | $C$ | $C$ | 11 | 157924.64 | 4681.38 | -78951.32 |

Note: Full model was used in Fig 2. C and t represent cluster membership and time, respectively. AICc is the sample corrected Akaike Information Criterion, Delta AICc is the change in AICc from the full model, and LL is the log-likelihood estimate.

# Effect of latent pain trajectories on 30-day and 1-year postsurgical pain

We used separate multiple logistic regression models to ascertain the relationships between acute pain trajectories and self-reported pain at 30-days and 1-year after surgery. We dichotomized pain scores at baseline, 30-days and 1-year as either moderate/severe pain (score ≥3) or minimal pain (score<3). The logistic model incorporated as covariates the baseline characteristics of each patient. Backward selection was used to remove covariates deemed insignificant (p < 0.05) from the model unless their inclusion modified the estimated effect substantially. The complete set of inferential results of both 30-day and 1-year models are provided in Table A3.

**Table A3. Effects of Latent Pain Trajectories on 30-day and 1-year Pain.**

|  | **30-Day Clinically Significant Pain** | | | **1-Year Clinically Significant Pain** | | |
| --- | --- | --- | --- | --- | --- | --- |
| *Predictors* | *Odds Ratios* | *95% CI* | *p-value* | *Odds Ratios* | *95% CI* | *p-value* |
| (Intercept) | 0.27 | 0.15 – 0.49 | **<0.001** | 0.58 | 0.23 – 1.46 | 0.250 |
| Cluster^a^ |  |  |  |  |  |  |
| II | 0.67 | 0.51 – 0.89 | **0.005** | 0.86 | 0.62 – 1.20 | 0.380 |
| III | 0.74 | 0.56 – 0.99 | **0.041** | 0.87 | 0.61 – 1.23 | 0.421 |
| IV | 0.46 | 0.26 – 0.82 | **0.009** | 0.80 | 0.43 – 1.50 | 0.493 |
| Age | 0.99 | 0.98 – 1.00 | 0.093 | 0.99 | 0.98 – 1.00 | **0.028** |
| Female | 1.21 | 0.96 – 1.52 | 0.099 |  |  |  |
| Race^b^ |  |  |  |  |  |  |
| Black or African American | 1.10 | 0.73 – 1.66 | 0.654 |  |  |  |
| Native Hawaiian or other Pacific Islander | 0.24 | 0.03 – 1.91 | 0.176 |  |  |  |
| Smoking^c^ |  |  |  |  |  |  |
| smoking [1] | 1.55 | 1.03 – 2.32 | **0.035** | 1.45 | 0.91 – 2.31 | 0.118 |
| smoking [2] | 1.35 | 1.05 – 1.72 | **0.017** | 1.28 | 0.96 – 1.70 | 0.094 |
| Charlson Index^d^ |  |  |  |  |  |  |
| CCI = 1 | 1.47 | 1.10 – 1.98 | **0.010** | 0.97 | 0.68 – 1.37 | 0.853 |
| CCI = 2 | 1.07 | 0.83 – 1.37 | 0.614 | 0.78 | 0.57 – 1.06 | 0.113 |
| Regional anesthesia | 0.77 | 0.55 – 1.09 | 0.137 |  |  |  |
| Moderate/severe baseline pain | 3.28 | 2.63 – 4.09 | **<0.001** | 3.01 | 2.31 – 3.92 | **<0.001** |
| Surgical Category^e^ |  |  |  |  |  |  |
| Cardiothoracic |  |  |  | 0.69 | 0.31 – 1.57 | 0.380 |
| MIS |  |  |  | 0.18 | 0.07 – 0.48 | **0.001** |
| Orthopedic |  |  |  | 0.37 | 0.19 – 0.73 | **0.004** |
| Other |  |  |  | 0.37 | 0.19 – 0.70 | **0.003** |
| Vascular |  |  |  | 0.47 | 0.19 – 1.16 | 0.102 |
| Notes: a: Overall variable effect had p=.007 at 30 days and p=.79 at 1 year; b: p=.24; c: p=.016 and p=.12; d: p=.03 and p=.24; e: p<.0001. P-values based on Type III likelihood ratio Chi-square tests. | | | | | | |

# Evaluation of logistic regression models

We evaluated the fit and predictive power of each logistic model. The model fit was assessed using the Hosmer-Lemeshow goodness-of-fit test. The results show that the both models exhibited reasonable fit, that is, no significant deviation from the null scenario of a good fitting model (30-day p-value=0.34; 1-year p-value=0.91). The predictive power of the models was assessed using receiver operating characteristic (ROC) curves of sensitivity versus 1-specificity. The areas under the curves (AUCs) were reported along with the suggested probability threshold (see Fig A3). The models have AUCs equal to 0.68 and 0.67, respectively.


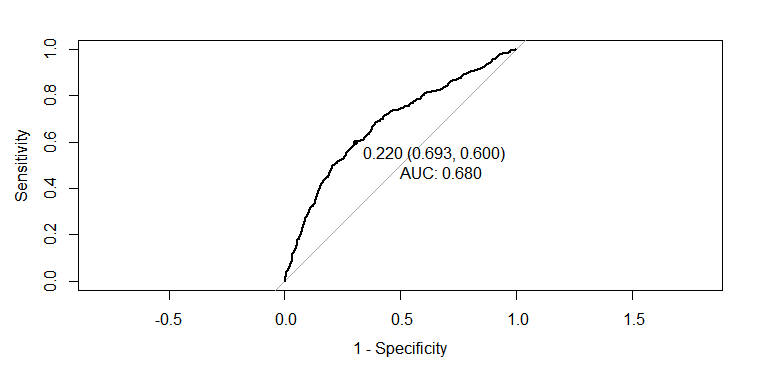

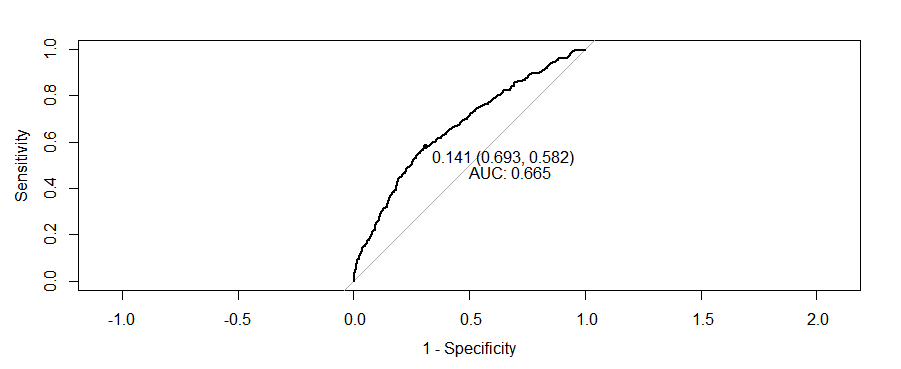


**Fig A3. ROC Curves of Logistic Regression Models for 30-day (left) and 1-year (right) Moderate/Severe Pain.**

# Further evaluation of data collection

At the suggestion of a referee, we investigated the question of whether reported pain levels impacted the frequency of data collection. For instance, it is possible that patients who reported higher levels of pain would be monitored more closely and thus have more observations. A crude assessment of the relationship between maximum reported pain and the number of observations showed a pronounced positive correlation. However, when we examined the daily number of observation by subject versus their maximum pain (Fig A4), we observed far greater homogeneity in how often they report pain. On the other hand, patient pain scores were reported less frequently in days 3 and 4 compared to days 1 and 2. Coupled with the results in Table 1 and Fig 3 that Cluster 3 had far fewer patients, this suggests that the relationship between maximum pain and number of observations is strongly confounded by the fact that patients with higher pain levels are likely to stay hospitalized longer and, when this is taken into account, pain levels have a minor impact on observation frequency.


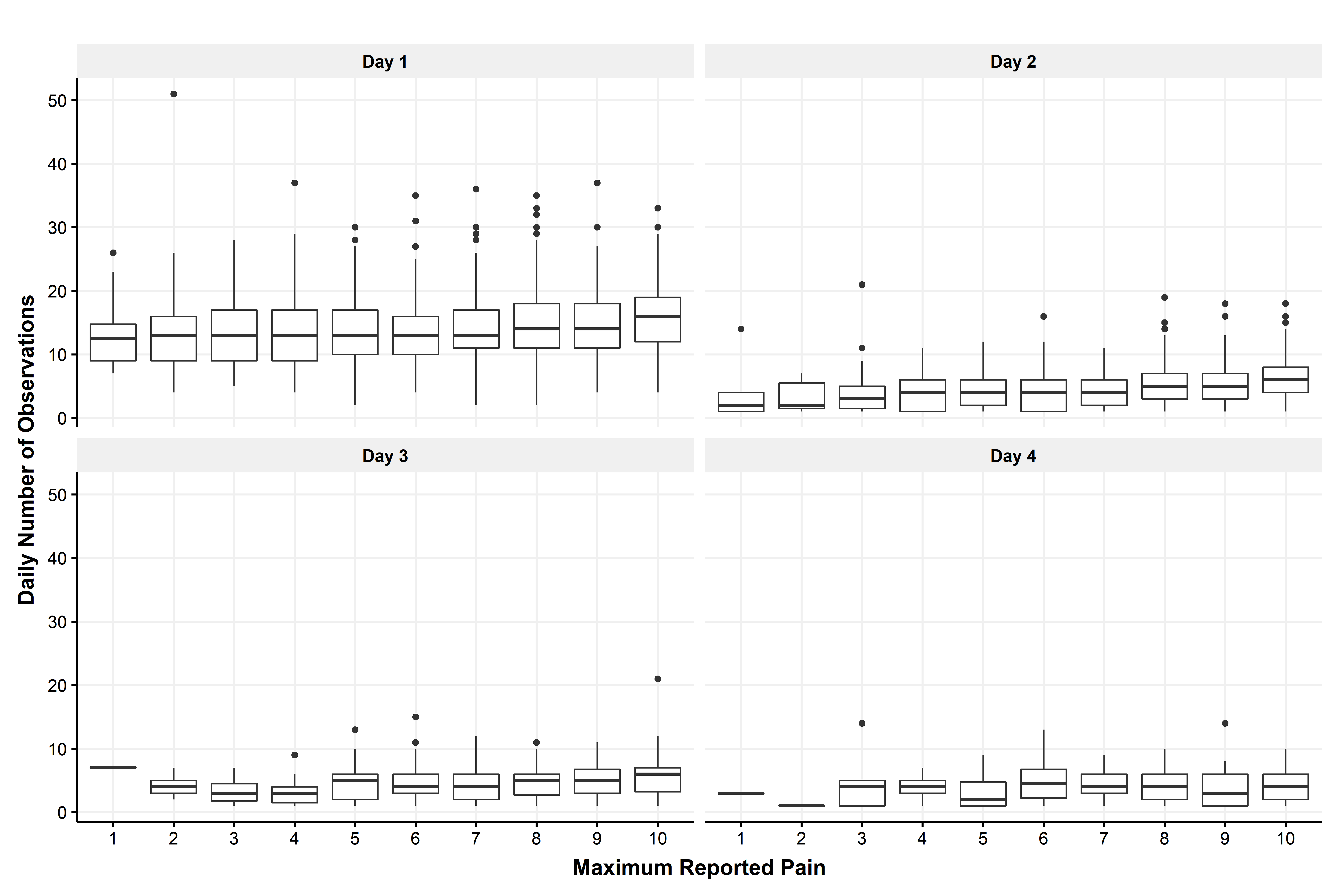


**Fig A4: Daily Frequency of Observations by Maximum Experienced Pain.**

# Assessment of clustering and prognostic models using resampling

In this section, we outline a resampling approach to evaluating the entire process of identifying latent trajectory clusters of pain, and then using those as predictors of long-term pain at 30-days and 1-year. We conducted an analysis akin to cross-validation were sub-samples of the data were drawn randomly and for each sub-sample we first identified the clusters and used them to predict long-term pain after adjusting for the same covariates as in the original model based on the full data. Because cluster designations were not identifiable, we looked at the models’ ability to predict long-term pain instead of the coefficients of each cluster. The objective is to determine whether the predictive power is consistent even if it’s based on a smaller set of observations. For this analysis we used 10 random samples of 75% of the data and for each sample used the *traj* method to identify clusters, and then used those clusters to predict pain at 30-days and 1-year. For each resulting model we plotted the ROC curves given in Fig A3. The results show that the predictive strength of each model is homogenous across different sub-samples of the data.


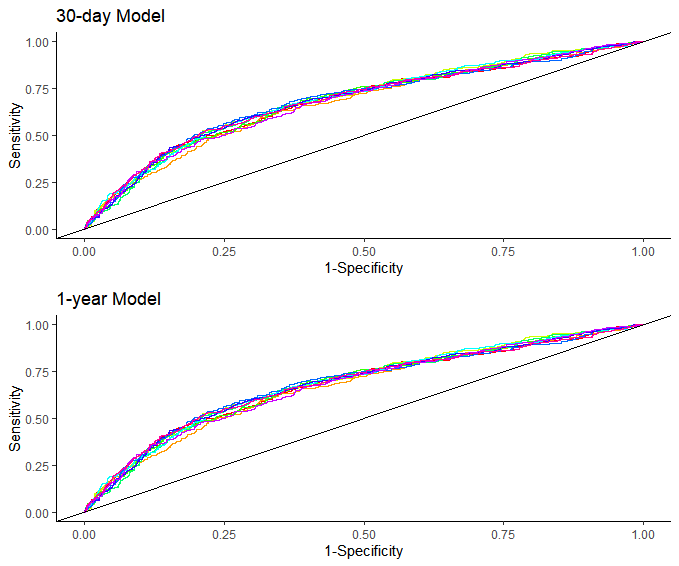


Fig A4: Analysis of the prediction sensitivity and specificity for models based on 10 random sub-samples of the data.

# References

1. Leffondré, K., Abrahamowicz, M., Regeasse, A., Hawker, G.A., Badley, E.M., McCusker, J., and Belzile, E., *Statistical measures were proposed for identifying longitudinal patterns of change in quantitative health indicators.* Journal of clinical epidemiology, 2004. **57**(10): p. 1049-1062.

2. Sylvestre, M.-P., McCusker, J., Cole, M., Regeasse, A., Belzile, E., and Abrahamowicz, M., *Classification of patterns of delirium severity scores over time in an elderly population.* International Psychogeriatrics, 2006. **18**(4): p. 667.

3. RCore, T., *R: a language and environment for statistical computing. R Foundation for Statistical Computing, Vienna, Austria*. 2020.

4. Plonsky, L., *Advancing quantitative methods in second language research*. 2015: Routledge.

5. Sellers, K.F. and Shmueli, G., *A flexible regression model for count data.* The Annals of Applied Statistics, 2010: p. 943-961.

6. Hall, D.B., *Zero‐inflated Poisson and binomial regression with random effects: a case study.* Biometrics, 2000. **56**(4): p. 1030-1039.

7. Brooks, M.E., Kristensen, K., van Benthem, K.J., Magnusson, A., Berg, C.W., Nielsen, A., Skaug, H.J., Machler, M., and Bolker, B.M., *glmmTMB balances speed and flexibility among packages for zero-inflated generalized linear mixed modeling.* The R journal, 2017. **9**(2): p. 378-400.
